# *Haemophilus ducreyi* Cutaneous Ulcer Strains Diverged from Both Class I and Class II Genital Ulcer Strains: Implications for Epidemiological Studies

**DOI:** 10.1371/journal.pntd.0005259

**Published:** 2016-12-27

**Authors:** Dharanesh Gangaiah, Stanley M. Spinola

**Affiliations:** 1 Departments of Microbiology and Immunology, Indiana University School of Medicine, Indianapolis, Indiana, United States of America; 2 Department of Medicine, Indiana University School of Medicine, Indianapolis, Indiana, United States of America; 3 Departments of Pathology and Laboratory Medicine, Indiana University School of Medicine, Indianapolis, Indiana, United States of America; University of Tennessee, UNITED STATES

## Abstract

**Background:**

*Haemophilus ducreyi* has emerged as a major cause of cutaneous ulcers (CU) in yaws-endemic regions of the tropics in the South Pacific, South East Asia and Africa. *H*. *ducreyi* was once thought only to cause the genital ulcer (GU) disease chancroid; GU strains belong to 2 distinct classes, class I and class II. Using whole-genome sequencing of 4 CU strains from Samoa, 1 from Vanuatu and 1 from Papua New Guinea, we showed that CU strains diverged from the class I strain 35000HP and that one CU strain expressed β-lactamase. Recently, the Center for Disease Control and Prevention released the genomes of 11 additional CU strains from Vanuatu and Ghana; however, the evolutionary relationship of these CU strains to previously-characterized CU and GU strains is unknown.

**Methodology/Principal Findings:**

We performed phylogenetic analysis of 17 CU and 10 GU strains. Class I and class II GU strains formed two distinct clades. The class I strains formed two subclades, one containing 35000HP and HD183 and the other containing the remainder of the class I strains. Twelve of the CU strains formed a subclone under the class I 35000HP subclade, while 2 CU strains formed a subclone under the other class I subclade. Unexpectedly, 3 of the CU strains formed a subclone under the class II clade. Phylogenetic analysis of *dsrA*-*hgbA*-*ncaA* sequences yielded a tree similar to that of whole-genome phylogenetic tree.

**Conclusions/Significance:**

CU strains diverged from multiple lineages within both class I and class II GU strains. Multilocus sequence typing of *dsrA*-*hgbA*-*ncaA* could be reliably used for epidemiological investigation of CU and GU strains. As class II strains grow relatively poorly and are relatively more susceptible to vancomycin than class I strains, these findings have implications for methods to recover CU strains. Comparison of contemporary CU and GU isolates would help clarify the relationship between these entities.

## Author Summary

Cutaneous ulcers (CU) in children in yaws-endemic regions have long been attributed to *Treponema pallidum* subsp. *pertenue*; however, recent studies show that *Haemophilus ducreyi* is an important cause of CU in these regions. *H*. *ducreyi* was once thought to cause only the genital ulcer (GU) disease chancroid; phylogenetically, GU strains belong to two distinct classes called class I and class II. We previously showed that CU strains obtained from Samoa, Vanuatu and Papua New Guinea are genetically almost identical to class 1 GU strains. In this study, using published genomes from 11 additional CU strains from Ghana and Vanuatu, we show that CU strains diverged from both class I and class II GU strains and that multiple CU clones may circulate in endemic areas. These findings have implications for epidemiological typing and recovery of *H*. *ducreyi* strains from both CU and GU clinical samples.

## Introduction

*Haemophilus ducreyi* causes chancroid, a sexually transmitted disease that manifests as genital ulcers (GU) and regional lymphadenitis in adults. Due to syndromic management of genital ulcers and lack of surveillance, the current global prevalence of chancroid is undefined but has declined over the last decade in many former endemic areas [1]. Phylogenetically, GU strains belong to 2 distinct groups called class I and class II, which differ in their expression of several surface proteins and lipooligosaccharide and in their susceptibility to vancomycin and diverged from each other approximately 1.95 million years ago [2–7].

In addition to chancroid, recent studies conducted in the yaws-endemic regions of the South Pacific islands and equatorial Africa show that *H*. *ducreyi* has emerged as an important cause of nonsexually transmitted cutaneous ulcers (CU) in children [1, 8–12]. In studies conducted in yaws-endemic villages on Vanuatu and Lihir Island of Papua New Guinea, *H*. *ducreyi* was detected in 39% to 60% of all skin ulcers, while *Treponema pallidum* subspecies *pertenue*, the etiologic agent of yaws, which was thought to be the major cause of CU, was detected in 15% to 34% of skin ulcers, respectively [8, 10, 11]. In Ghana and the Solomon Islands, *H*. *ducreyi* DNA was detected in 9% to 32% of CU, and no other pathogen DNA was detected [1, 9, 12]. The reasons for the variation in the sensitivities of the PCR-based tests in these studies are unclear; but the data suggest that a substantial proportion of cases of CU may be caused by organism(s) that are yet to be identified.

Using whole-genome sequencing and evolutionary analyses of 4 CU strains from Samoa, 1 from Vanuatu and 1 from Papua New Guinea, we previously showed that CU strains are almost genetically identical to the class I strain 35000HP and that CU strains form a subcluster within the class I clade of *H*. *ducreyi* [7, 13]. These studies were limited by small sample size and lack of samples from other endemic regions. The Center for Disease Control and Prevention recently released genomes of 11 additional CU strains, 6 from Ghana and 5 from Vanuatu [14]. However, the relationship of these CU strains to previously-characterized CU and GU strains is not known. In the present study, we performed phylogenetic analyses of all the available CU and GU strains whose genomes have been released. We also examined the utility of a multilocus sequence typing system developed by Humphreys and coworkers to classify the strains [6, 15]. As we had done previously [7], we also analyzed the genomes of the recently described CU strains for the presence of acquired antimicrobial resistance genes and genes required for the virulence of 35000HP in experimentally infected human volunteers.

## Materials and Methods

The genomes of 11 uncharacterized CU strains (GenBank accession no. CP015424 to CP015434) and 6 previously-characterized CU and 10 GU strains (GenBank accession no. CP011218 to CP011231) were downloaded from GenBank and used in the present study for phylogenetic analyses [7, 13, 14]. The genome of 35000HP (GenBank accession no. NC_002940.2), which has been well-characterized in the human challenge studies, was used as the reference strain for all analyses in this study. The genomes were aligned using progressiveMauve [16]. Whole-genome alignments were imported into Mega 7, manually edited for accuracy and subjected to model testing to identify the best-fit model of nucleotide substitution [17]. Using the best fit model (Hasegawa-Kishino-Yano plus invariant sites plus gamma-distributed model), a maximum likelihood tree was generated with 500 bootstrap replicates. Phylogenetic analyses were also performed on concatenated sequences of *dsrA*, *hgbA* and *ncaA*, which have been previously used for multilocus sequence-based epidemiological investigation of *H*. *ducreyi* strains [6]. As previously described [7], the uncharacterized CU genomes were searched for known *H*. *ducreyi* virulence genes using Basic Local Alignment Search Tool and for acquired antimicrobial resistance genes using ResFinder [18].

## Results

**Fig 1:**
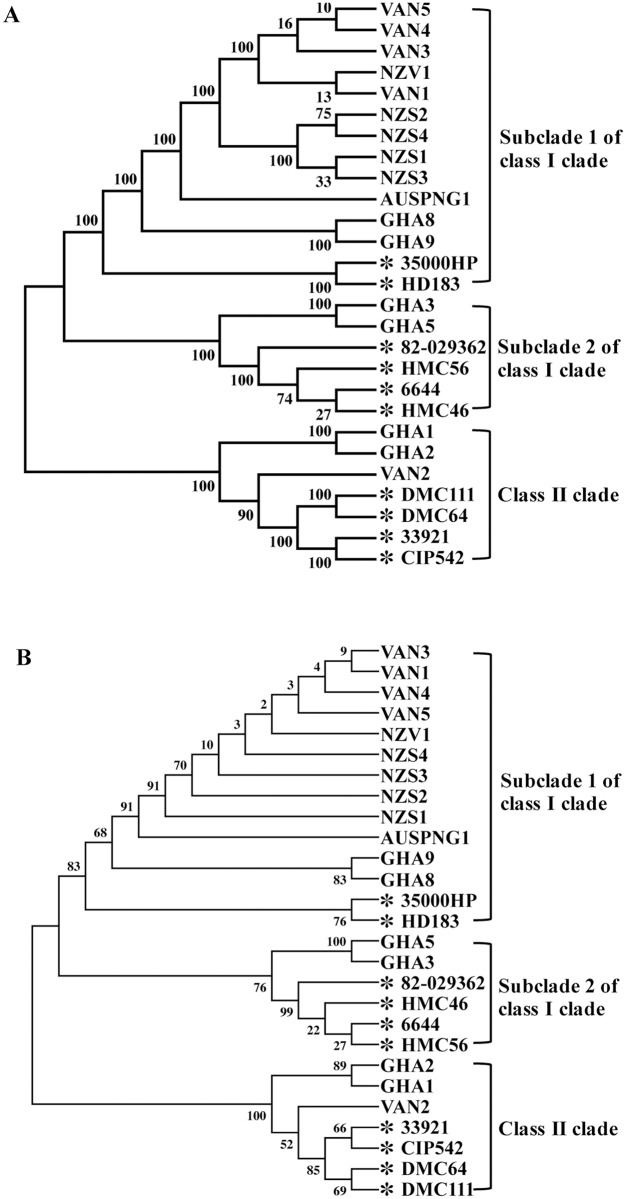
The evolutionary relationship of the uncharacterized CU strains to previously-characterized CU and GU strains. **A**. Phylogenetic tree of *H*. *ducreyi* CU and GU strains based on whole-genome sequences. **B**. Phylogenetic tree of *H*. *ducreyi* CU and GU strains based on *dsrA*-*hgbA*-*ncaA* sequences. The evolutionary relationship was inferred by using the Maximum Likelihood method based on the Hasegawa-Kishino-Yano model. The reliability of the tree was tested using 500 bootstrap replicates; the bootstrap support values are indicated next to the branches in percentage. Asterisks, GU strains. Strain designations for the CU strains included their country of origin as follows: Vanuatu, VAN, NZV; Samoa, NZS; Papua New Guinea, AUSPNG and Ghana, GHA. As reported previously [7], the GU strains have a worldwide distribution.

As reported previously [7], whole-genome phylogenetic analyses showed that class I and class II GU strains formed two distinct clades (Fig 1A). In this analysis, the class I GU strains formed two subclades with one containing 35000HP and HD183 (subclade 1 in Fig 1A) and the other containing the remainder of the class I strains (subclade 2 in Fig 1A). The 6 previously-characterized CU strains and 6 of the uncharacterized CU strains from Vanuatu and Ghana formed a subclone that diverged from the class I 35000HP subclade; all the Vanuatu strains and all the Samoan strains formed separate groups within this subclone (Fig 1A). Two of the Ghanaian strains diverged from the other class I subclade (Fig 1A). Unexpectedly, 3 strains from Vanuatu and Ghana formed a subclone under the class II strains (Fig 1A).

Previously, *dsrA*, *hgbA* and *ncaA* were used for multilocus sequence typing of CU and GU strains of *H*. *ducreyi* [6, 15]. Except for minor changes in branch positions within the class I subclades and class II clade, phylogenetic analysis of concatenated *dsrA*, *hgbA* and *ncaA* sequences from the 17 CU strains and 10 GU strains yielded a tree similar to that of whole-genome phylogenetic tree (Fig 1B). This finding confirms the reliability of *dsrA*, *hgbA* and *ncaA* genes for multilocus sequence typing of both class I and class II CU strains in endemic areas.

We had previously shown that CU strains contained no acquired antimicrobial resistance genes except for AUSPNG1, which expressed β-lactamase [7, 13]. Search for the presence of the acquired antimicrobial resistance genes in the genomes of recently reported CU strains showed that 4 of these strains (GHA3, GHA5, GHA8 and GHA9) contained *tet(B)*, which confers resistance to tetracycline, and 2 (GHA1 and GHA2) contained *catS*, which confers resistance to chloramphenicol; both of these resistance determinants are common in GU isolates [19]. None of the strains contained *bla* determinants. As had been reported for CU strains previously [7], search for known *H*. *ducreyi* virulence determinants in the genomes of the uncharacterized CU strains showed that they also contained all the virulence genes required for infection by strain 35000HP in the human challenge model (Table 1) [20].

**Table 1:** Comparison of the virulence genes of the uncharacterized CU strains to that of 35000HP and CIP542.

	Class I CU strains								Class II CU strains		
	VAN1	VAN3	VAN4	VAN5	GHA3	GHA5	GHA8	GHA9	VAN2	GHA1	GHA2
***cpxA***	35000HP	35000HP	35000HP	35000HP	35000HP	35000HP	35000HP	35000HP	CIP542	CIP542	CIP542
csrA*	35000HP	35000HP	35000HP	35000HP	35000HP	35000HP	35000HP	35000HP	35000HP	35000HP	35000HP
***dksA***	35000HP	35000HP	35000HP	35000HP	35000HP	35000HP	35000HP	35000HP	CIP542	CIP542	CIP542
***dltA***	35000HP	35000HP	35000HP	35000HP	35000HP	35000HP	35000HP	35000HP	CIP542	CIP542	CIP542
***dsrA***	A1	A1	A1	A1	A2	A2	A3	A3	B	B	B
***fgbA***	35000HP	35000HP	35000HP	35000HP	A	A	35000HP	35000HP	CIP542	CIP542	CIP542
***flp1***	A	A	A	A	A	A	A	A	CIP542	CIP542	CIP542
***flp2***	A	A	A	A	A	A	A	A	CIP542	CIP542	CIP542
***flp3***	A	A	A	A	A	A	A	A	CIP542	CIP542	CIP542
***hfq***	35000HP	35000HP	35000HP	35000HP	A	A	35000HP	35000HP	CIP542	CIP542	CIP542
***hgbA***	35000HP	35000HP	35000HP	35000HP	35000HP	35000HP	35000HP	35000HP	CIP542	CIP542	CIP542
***lspA1***	A	A	A	A	A	A	A	A	-	-	-
***lspA2***	A	A	A	A	A	A	A	A	-	-	-
luxS*	A	A	A	A	A	A	A	A	CIP542	CIP542	CIP542
***ncaA***	A	A	A	A	A	A	35000HP	35000HP	CIP542	CIP542	CIP542
***pal***	35000HP	35000HP	35000HP	35000HP	35000HP	35000HP	35000HP	35000HP	CIP542	CIP542	CIP542
***relA***	A	A	A	A	A	A	A	A	CIP542	CIP542	CIP542
***sapA***	35000HP	35000HP	35000HP	35000HP	35000HP	35000HP	35000HP	35000HP	CIP542	CIP542	CIP542
***sapB***	35000HP	35000HP	35000HP	35000HP	35000HP	35000HP	35000HP	35000HP	CIP542	CIP542	CIP542
***sapC***	A	A	A	A	A	A	A	A	CIP542	CIP542	CIP542
***spoT***	35000HP	35000HP	35000HP	35000HP	A	A	A	A	CIP542	CIP542	CIP542
***tadA***	35000HP	35000HP	35000HP	35000HP	A	A	35000HP	35000HP	CIP542	CIP542	CIP542
***wecA***	35000HP	35000HP	35000HP	35000HP	35000HP	35000HP	35000HP	35000HP	CIP542	CIP542	CIP542

35000HP, the nucleotide sequence is identical to that of 35000HP.

CIP542, the nucleotide sequence is identical to that of CIP542.

*, the *csrA* and *luxS* alleles of 35000HP and CIP542 are identical

A, the nucleotide sequence differs from 35000HP by at least 1 nucleotide but is identical within the class I CU strains; A1, A2, and A3 designate groups of strains with *dsrA* alleles that also differ from each other.

B, the nucleotide sequence is different from CIP542 by at least 1 nucleotide but is identical within the class II CU strains.

-, complete sequence not available for CIP542 and therefore, no comparison with the class II CU strains.

## Discussion

*H*. *ducreyi* was once thought to only cause the sexually transmitted genital ulcer disease chancroid in adults. However, recent studies show that *H*. *ducreyi* is an important cause of nonsexually transmitted cutaneous ulcers in children in tropics in the South Pacific, South East Asia and Africa [1, 8–12]. Previous whole-genome sequencing of 6 CU strains from Samoa, Vanuatu and Papua New Guinea showed that these CU strains diverged from class I GU strains [7, 13]. Phylogenetic analysis of the genomes of 11 recently reported CU strains [14] from Ghana and Vanuatu showed that CU strains diverged from both class I and class II GU strains and suggest that multiple CU clones may circulate in endemic areas. These findings have two implications: 1) Culture techniques may need to be modified to recover Class II strains. Relative to archived Class I GU strains, archived Class II GU strains grow poorly on media lacking antibiotics [2, 5] and exhibit larger zones of inhibition around vancomycin-impregnated disks (Tricia Humphreys, personal communication). The standard media used for isolation of *H*. *ducreyi* from clinical samples contains vancomycin [21, 22]. To isolate Class II strains, the incubation period of primary cultures may need to be extended beyond the standard 48 h used to recover *H*. *ducreyi* [22]. If vancomycin-susceptible strains are suspected [23], additional use of unsupplemented media may be considered, with the caveat that this would double the cost of cultures and may prove to be impractical in resource-poor areas [24] 2) The phylogenetic tree based on *dsrA*-*hgbA*-*ncaA* sequences is similar to that based on the whole-genome sequences. Therefore, *dsrA*-*hgbA*-*ncaA*-based multilocus sequence typing could be reliably used for epidemiological investigation of CU and GU strains.

The GU strain (35000HP) is highly infectious when experimentally inoculated into the skin of the upper arm of adults, with an estimated infectious dose of as few as 1 CFU [20]. The CU strains that form a subclone of the 35000HP branch are nearly genetically identical to 35000HP, differing by ~ 400 SNPs, most of which are synonymous, and express all genes known to be required for pustule formation for strain 35000HP [7]. These data raise the possibilities that GU strains have the biological potential to cause CU and that CU strains have the biological potential to cause GU. Before the implementation of yaws elimination campaigns in the early 1950s, yaws clearly occurred in many chancroid-endemic countries [25]. Yaws—a possible surrogate for *H*. *ducreyi*-associated CU—recently has been reported almost exclusively from countries that report no diagnostic data on chancroid [1, 25]. With the exception of the Central African Republic and Ghana, which report a 0.7% prevalence of chancroid in patients with GU [26], chancroid recently has been reported only in countries in which yaws is not thought to be endemic [1, 25]. This could mean that different routes of inoculation—sexual transmission for chancroid and nonsexual transmission for CU—have served to isolate GU and CU *H*. *ducreyi* strains into their respective anatomic compartments and adult and pediatric populations. Due to syndromic management of GU, we know of no recent *H*. *ducreyi* GU isolates available for characterization; a limitation of this study is that the CU strains were not compared to contemporaneous GU strains. In addition, a limitation of the literature is that no studies have simultaneously addressed the prevalence of chancroid and *H*. *ducreyi*-associated CU in the same region. Thus, the third implication of our analysis is that such studies are needed to understand the epidemiological relationship, if any, between currently circulating CU and GU strains.
